# Immortal time bias for life-long conditions in retrospective observational studies using electronic health records

**DOI:** 10.1186/s12874-022-01581-1

**Published:** 2022-03-27

**Authors:** Freya Tyrer, Krishnan Bhaskaran, Mark J. Rutherford

**Affiliations:** 1 Department of Health Sciences (Biostatistics Research Group), University of Leicester, Leicester, UK; 2 Department of Non-communicable Disease Epidemiology, London School of Hygiene & Tropical Medicine, London, UK

**Keywords:** Bias (epidemiology), Epidemiologic methods, Immortal time bias, Electronic health records, Life expectancy, Observational studies

## Abstract

**Background:**

Immortal time bias is common in observational studies but is typically described for pharmacoepidemiology studies where there is a delay between cohort entry and treatment initiation.

**Methods:**

This study used the Clinical Practice Research Datalink (CPRD) and linked national mortality data in England from 2000 to 2019 to investigate immortal time bias for a specific life-long condition, intellectual disability. Life expectancy (Chiang’s abridged life table approach) was compared for 33,867 exposed and 980,586 unexposed individuals aged 10+ years using five methods: (1) treating immortal time as observation time; (2) excluding time before date of first exposure diagnosis; (3) matching cohort entry to first exposure diagnosis; (4) excluding time before proxy date of inputting first exposure diagnosis (by the physician); and (5) treating exposure as a time-dependent measure.

**Results:**

When not considered in the design or analysis (Method 1), immortal time bias led to disproportionately high life expectancy for the exposed population during the first calendar period (additional years expected to live: 2000–2004: 65.6 [95% CI: 63.6,67.6]) compared to the later calendar periods (2005–2009: 59.9 [58.8,60.9]; 2010–2014: 58.0 [57.1,58.9]; 2015–2019: 58.2 [56.8,59.7]). Date of entry of diagnosis (Method 4) was unreliable in this CPRD cohort. The final methods (Method 2, 3 and 5) appeared to solve the main theoretical problem but residual bias may have remained.

**Conclusions:**

We conclude that immortal time bias is a significant issue for studies of life-long conditions that use electronic health record data and requires careful consideration of how clinical diagnoses are entered onto electronic health record systems.

**Supplementary Information:**

The online version contains supplementary material available at 10.1186/s12874-022-01581-1.

## Background

Electronic health records are increasingly being used to conduct real-world observational studies to determine the association between a treatment or exposure and outcome. However, such studies are prone to a number of biases. In particular, immortal time bias is a recognised limitation of observational studies [1–3] that has come to the forefront in recent years owing to the increasing complexity of observational cohort designs [4–8]. This bias occurs where there is a period of time during follow up where an event or death cannot occur [4]. It is often discussed in the context of pharmacoepidemiology where a delay between entering the study and being allocated a given therapy at baseline creates an ‘immortal’ period for the subject thereby creating an apparent advantage for the group that is given the therapy. In these types of study, immortal time bias can generally be reduced, if not eliminated completely, by adapting the analysis; for example, through application of prescription time-distribution matching (PTDM), time-dependent or sequential Cox approaches, or landmark analyses [9–11].

However, immortal time bias is not specific to pharmacoepidemiology studies and can represent a significant problem where the exposure is a life-long condition or disability, defined here by a long-term condition that typically starts before adulthood and cannot, at present, be cured. This is a particular issue for electronic health record studies where there is a period of delay between onset and diagnosis of a given condition during which the individual is effectively ‘immortal’. We demonstrate this using an example of electronic health record data of life expectancy among patients with and without intellectual disabilities from the UK Clinical Practice Research Datalink (CPRD). Primary care incentives in England to identify people with intellectual disabilities between 2004 and 2008 (and 2014 for young adults/children) [12–16] means that we might expect to see increases in diagnoses over time in patients who are already registered at a given surgery. We show that lack of consideration of immortal time bias in this population may lead us to draw incorrect conclusions about life expectancy across different calendar year periods. Our findings also have applicability to other long-term conditions with delays in diagnosis or prolonged latency periods.

## Methods

### Source of data

For this example, we used the Clinical Practice Research Datalink (CPRD GOLD), linked (person-level) with hospital episode statistics (HES) and death registrations from the Office for National Statistics (approved study protocol number: 19_267RA3). The CPRD is an electronic health record research database of more than 11.3 million patients, broadly representative of the national population in terms of age, gender, and ethnicity [17], from general practice (GP) surgeries in the UK – of which approximately 75% in England consent to linkage to deaths data. The study followed the Reporting of studies Conducted using Observational Routinely-collected health Data (RECORD) checklist [18] (see supplementary Table S1).

Diagnostic codes used in this study are reported in supplementary Table S2. The initial extract from the CPRD has been described previously [19] and was based on the following inclusion criteria: registered at the GP surgery at any time between 1 Jan 2000 to 29 Sept 2019; linkage data available; and 10 years old or over to account for delays in reporting of diagnoses of intellectual disability in children [20]. An additional 23 patients with Angelman or Cockayne syndrome were added in August 2021 after an amendment to the original protocol (approved March 2020 but delayed during the COVID period). A random sample of people without intellectual disabilities was used for the comparison group with the same eligibility criteria (but without a diagnosis of intellectual disability; please see Fig. S1 in the supplementary material for a data flow diagram). The initial extract included 33,867 people with intellectual disabilities ever in their records (the exposed population) and a random sample of 980,586 people (initially 1 million prior to exclusions) without intellectual disabilities (the unexposed population), although population sizes varied by the immortal time bias approach adopted. Baseline totals, age and observation period under the five approaches are shown in Table 1. Further baseline characteristics are shown in supplementary Table S3.

**Table 1:** Characteristics of the study population using different methods to handling immortal time bias

CHARACTERISTICS	EXPOSED		NON-EXPOSED	
	People with intellectual disabilities N (%) / median (range)		People without intellectual disabilities N (%) / median (range)	
**Method 1**				
Total	33,867	(100.00)	980,586	(100.00)
Age (years) at baseline:	29.0	(10–102)	34.0	(10–108)
Length of observation time (years)	6.5	(< 0.1–19.7)	5.0	(< 0.1–19.7)
**Method 2**				
Total	33,867	(100.00)	980,586	(100.00)
Age (years) at baseline:	31.0	(10–102)	34.0	(10–108)
Length of observation time (years)	4.6	(< 0.1–19.7)	5.0	(< 0.1–19.7)
**Method 3**^**a**^				
Total	33,867	(100.00)	338,670	(100.00)
Age (years) at baseline:	31.0	(10–102)	34.0	(10–108)
Length of observation time (years)	4.6	(< 0.1–19.7)	3.6	(< 0.1–19.7)
**Method 4**^**b**^				
Total	33,244	(100.00)	980,586	(100.00)
Age (years) at baseline:	33.0	(10–106)	34.0	(10–108)
Length of observation time (years)	2.2	(< 0.1–19.6)	5.0	(< 0.1–19.7)
**Method 5**^**c**^				
Total	33,867	(100.00)	991,879	(100.00)
Age (years) at baseline:	31.0	(10–102)	34.0	(10–108)
Length of observation time (years)	4.6	(< 0.1–19.7)	5.0	(< 0.1–19.7)

^a^*n* = 641,916 individuals from the unexposed population were discarded under Method 3 because they were not matched

^b^*n* = 623 individuals from the exposed population were excluded from this analysis under Method 4 because they entered on or after they were censored/died (i.e. system date linked to the diagnosis was after date of leaving/death)

^c^Individuals could contribute to both the exposed and unexposed populations under Method 5, as reflected in the baseline values

Date of entry into the cohort was defined as the latest date according to the person and practice’s characteristics: 01 Jan 2000; date of registration with the GP practice; date the practice was defined as being up to standard (using the CPRD’s own quality indicators); or date the individual turned 10 years old (to align with the eligibility criteria). Additional entry cohort criteria were specified according to the approach used. Date of exit from each calendar period was calculated in the same way throughout as: date of death; date of end of calendar period; date of last practice update (latest 29 Sep 2019); or date of transfer out of practice, whichever was first. If the patient died after their date of exit from the cohort, they were censored on the date of exit.

### Exposure/control definitions to handling immortal time bias under five different approaches

**Fig. 1:**
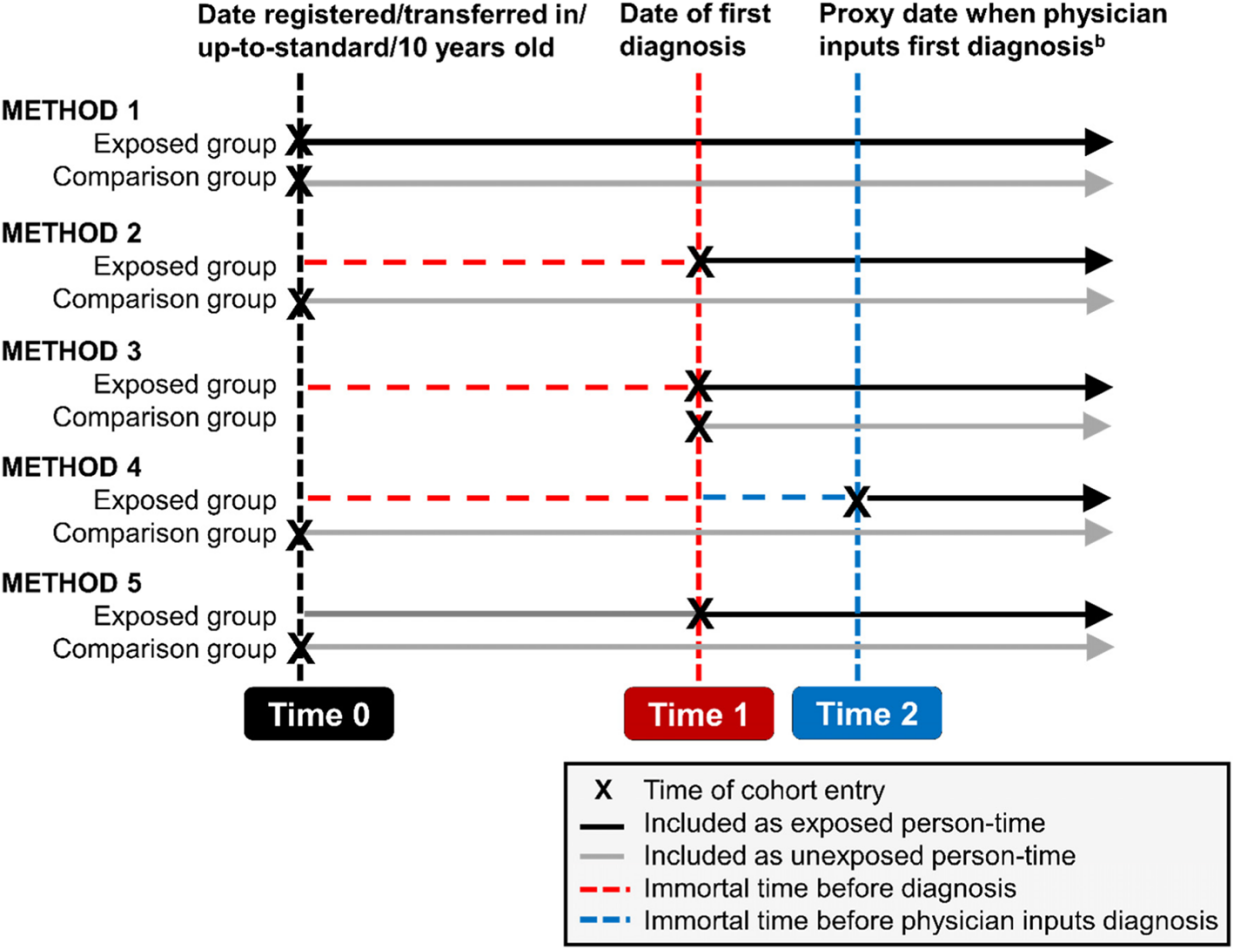
Diagram of exposed^a^ person-time under five methods for studies of life-long conditions using electronic health record data. ^a^ individuals with an intellectual disability diagnosis prior to registration/transfer (i.e. prevalent users in pharmacoepidemiology studies) entered the cohort as normal. ^b^ Time 2 represents the date that the clinician was assumed to have input the first intellectual disability diagnosis on their GP electronic health record database, given by the system date (‘sysdate’) attached to the diagnosis and diagnosis date

We present five approaches to defining cohort entry time when calculating life expectancy in people with and without intellectual disabilities and describe the impact that each approach has on life expectancy estimates in the context of immortal time bias. All methods involved changes in the handling of the exposed population. The second and third method also involved changes to the unexposed (control) population. The five methods compared are summarised in Fig. 1.

#### Method 1: treating immortal time as observation time

Method 1 (‘immortal time included’) involved applying no additional cohort entry criteria to either exposed or unexposed populations such that both populations are treated in the same way.

#### Method 2: excluding immortal time before date of first exposure diagnosis

Method 2 (‘immortal time excluded’) involved adding date of intellectual disability diagnosis to the entry criteria such that date of entry into the cohort was set to the date of intellectual disability diagnosis if this was *after* the entry criteria defined for Method 1 (Fig. 1). The comparison group patients entered at the date of registration/start of follow-up, as in Method 1. This approach has been described in pharmacoepidemiology studies in the context of excluding immortal time prior to treatment initiation in the treated group [10].

#### Method 3: matching cohort entry to first exposure diagnosis

The third approach (‘matched’) involved excluding immortal time, as in Method 2, but then matching the exposed individuals to unexposed individuals at a 1:10 ratio (chosen to maximise the number in the comparison group without losing controls) by date of cohort entry. This approach was designed to give a more balanced distribution of cohort entry date than Method 2 (in which entry dates for the comparison group tended to be earlier by design). The method is similar to the PTDM approach described for pharmacoepidemiology studies where dates of initiating therapy vary between treatment groups [10, 21, 22]. The difference between this and our approach is that PTDM involves differentiating between the ‘never treated’ and ‘ever treated’ groups such that cohort entry dates in the ‘never treated’ group are shifted to the date that the ‘ever treated’ group first started their treatment [10]. The approach, therefore, requires conditioning on the future and depends on length of the follow-up period since those in the ‘never treated’ group are allowed to move to the ‘ever treated’ group if the follow-up period is sufficiently long. Instead, our approach involved matching cohort entry dates against unexposed individuals, which included ‘never exposed’ individuals (i.e. without intellectual disabilities; PTDM approach) and ‘ever exposed’ individuals (i.e. people with intellectual disabilities *prior to* their first diagnosis). Therefore, individuals with intellectual disabilities could contribute person-time to both unexposed (prior to their first diagnosis) and exposed populations (after their first diagnosis).

Ten matches (random without replacement) for each exposed individual were initially selected from the pool of unexposed individuals who entered the study within 150 days (to balance follow-up time) prior to the index date (i.e. date of cohort entry for exposed individuals). Where all 10 matches could not be found within this time constraint (*n* = 24 patients), controls were selected from the pool of unexposed individuals who entered at any time prior to the index date and were still at risk. Dates of cohort entry in the unexposed population were then updated to the index date.

#### Method 4: excluding time before proxy date of inputting first exposure diagnosis

The fourth approach (‘proxy input date’) involved incorporating the date that the exposed population’s diagnosis was assumed to be input by the physician onto the GP surgery’s electronic health record system. This is not typically an issue with pharmacoepidemiology studies because the date of prescribing treatment is usually close to the date that the individual commences the treatment. However, for a life-long condition such as intellectual disabilities, physicians may choose to backdate the first exposure diagnosis to the patient’s date of birth. As well as diagnosis date, the CPRD provides a linked variable (‘system date’) for each diagnosis which can correspond to the date that the diagnosis was entered. However, this variable is also updated when a person transfers to the GP surgery, when there is an update in the GP software system used, or when the record is amended [23] which may lead to erroneous loss of person-years in the exposed group. To investigate this, date of entry was set to the date attached to the first intellectual disability diagnosis if it was later than the date of entry defined in Method 1 or Method 2 (by design; Fig. 1). In this method, the unexposed patients entered at the date of registration/start of follow-up period as in Method 1.

#### Method 5: treating exposure as a time-dependent measure

The final approach (‘time-dependent’) to handling immortal time bias involved treating the exposure as a time-dependent variable. This is perhaps the most common approach used in pharmacoepidemiology studies to control for immortal time bias and involves adapting the analysis so that individuals’ exposure to a therapy is allowed to change during the follow-up period [10, 22]. It can be used to investigate the effect of individual therapies where more than one is under investigation [24] and is also advocated as a means of avoiding immortal time bias caused by temporal variability in the onset of certain conditions, such as the menopause [3]. In this study, the approach involved allowing individuals in the exposed population to contribute to the unexposed population until their first intellectual disability diagnosis whereupon they started contributing to the exposed population (Fig. 1). The advantage of this method is that people with intellectual disabilities contribute person years in the same way as those who died before they had the opportunity to have an intellectual disability diagnosis.

### Statistical analyses

For the purposes of this work, data were split into the following calendar periods: 2000–2004; 2005–2009; 2010–2014; and 2015–2019.

To calculate life expectancy (additional life years expected to live) in both the exposed and unexposed populations, the Chiang’s abridged life table approach [25–28] was used. This approach has been described in detail elsewhere [28], but briefly involves stratifying by exposed and unexposed status and constructing a table of probabilities that individuals will survive in a defined age interval conditional on surviving to the start of that age interval. The product of probabilities is then used to calculate survival to each age interval and life expectancy is estimated as the cumulative number of years lived using information from all subsequent age intervals divided by the population at the start of the given age interval.

Confidence intervals for the derived life expectancies were calculated using the Chiang II approach as advocated by Eayres & Williams [29]. This involves adding a correction term to the original Chiang variance to account for the under-estimation of the ‘true’ variance at the last age interval by assuming variance is zero rather than basing the estimate on length of survival [30].

## Results

**Fig. 2:**
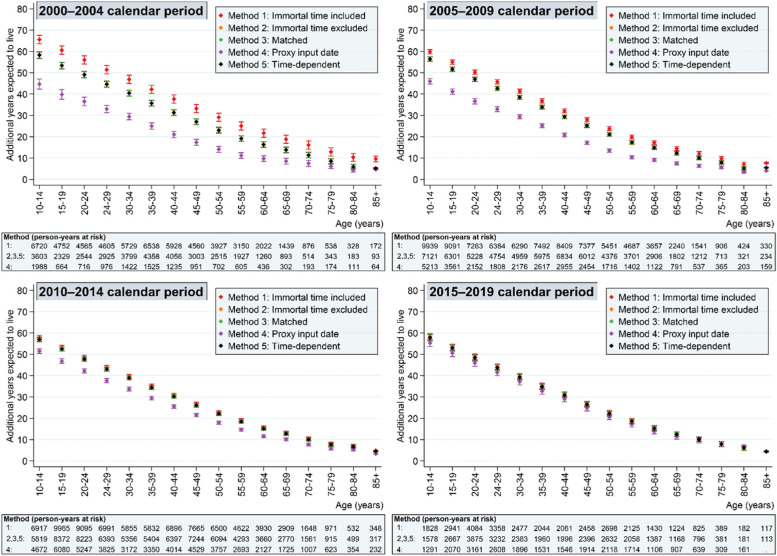
Life expectancy by calendar period in exposed individuals (people with intellectual disabilities) under the five methods to handling immortal time bias

**Fig. 3:**
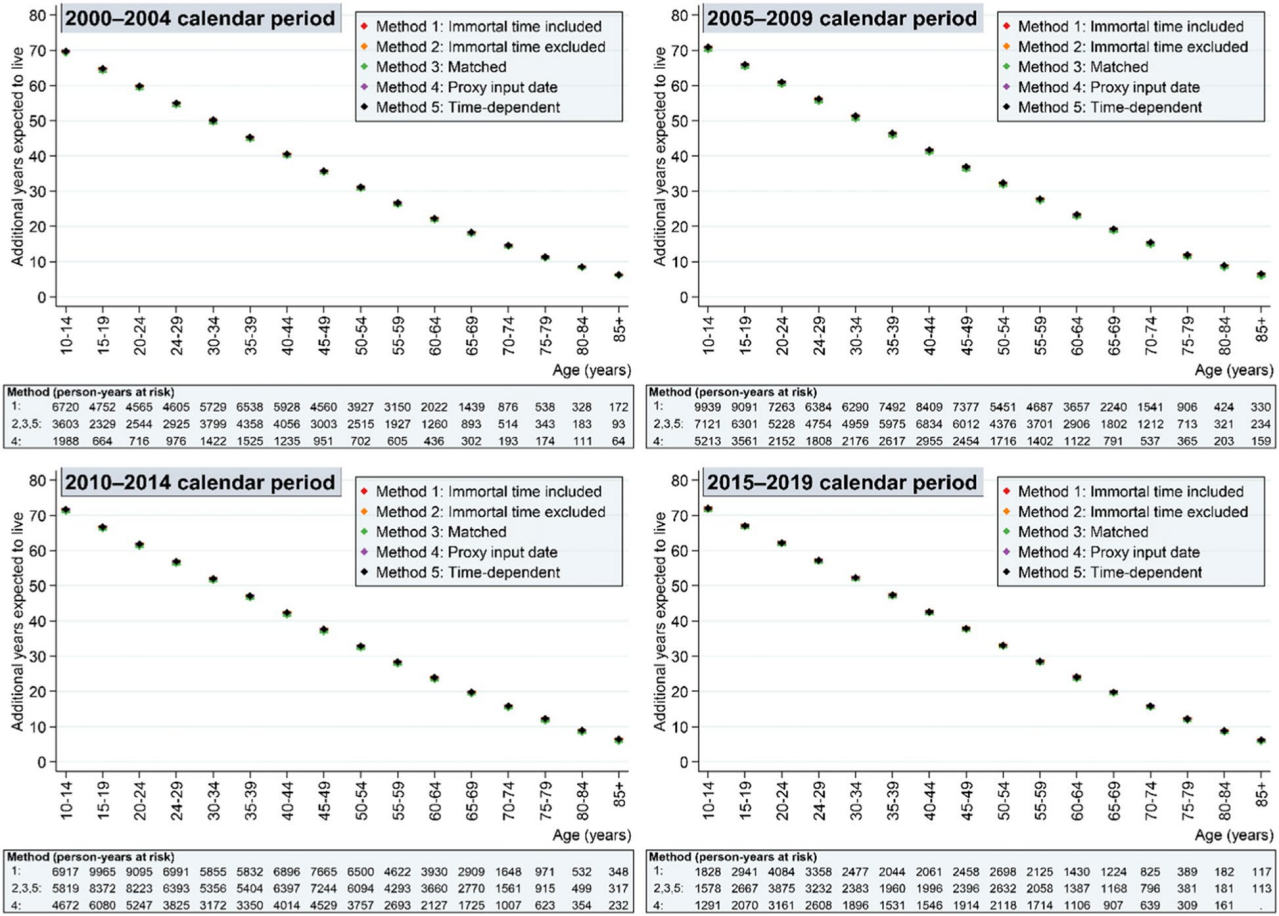
Life expectancy by calendar period in unexposed individuals (people without intellectual disabilities) under the five methods to handling immortal time bias

Figures 2 and Fig. 3 summarise the life expectancy findings for the exposed (Fig. 2) and unexposed (Fig. 3) population under the five methods. Each of the methods is described in more detail below.

### Method 1: treating immortal time as observation time

We can see from Fig. 2 that there is an apparent survival advantage in the first calendar period for the exposed population using Method 1, which is not observed in the unexposed population (Fig. 3). At age 10 years, for example, the estimated additional years expected to live in 2000–2004 was 65.6 (95% confidence interval [CI] 63.6,67.6) compared with 59.9 (58.8,60.9), 58.0 (57.1,58.9) and 58.2 (56.8,59.7) in the subsequent calendar periods. In the first calendar period and under Method 1, 38.5% of person-time (*n =* 21,506 person years) in the cohort with intellectual disabilities was *before* the first intellectual disability diagnosis (i.e. when a death could not occur), compared with 23.4, 9.1 and 4.8% in the second, third and fourth calendar periods respectively. Therefore, person-time was accrued for the exposed population during which a death could not occur.

### Method 2: excluding immortal time before date of first exposure diagnosis

Under Method 2, we can now see that the life expectancy advantages in the first calendar period are not as apparent. However, life expectancy remains slightly higher compared with the other calendar periods. This could be a real effect, but we speculate that some of the GP surgeries may have backdated intellectual disability diagnoses to date of birth during some key periods in response to policy initiatives in England (see discussion). In the first calendar period, 14.5% of person time (*n =* 8091 person years) had a backdated intellectual diagnosis to year of birth. This compares with 16.2 and 17.0% in the second and third calendar period during which many of the policy initiatives occurred and 14.6% in the final calendar period. We are unable to determine whether these records were updated when the patient was registered with the practice (when immortal time bias would not present a problem) or at some point during the registration period (see Method 4 [proxy input date] for one approach to handling this).

**Fig. 4:**
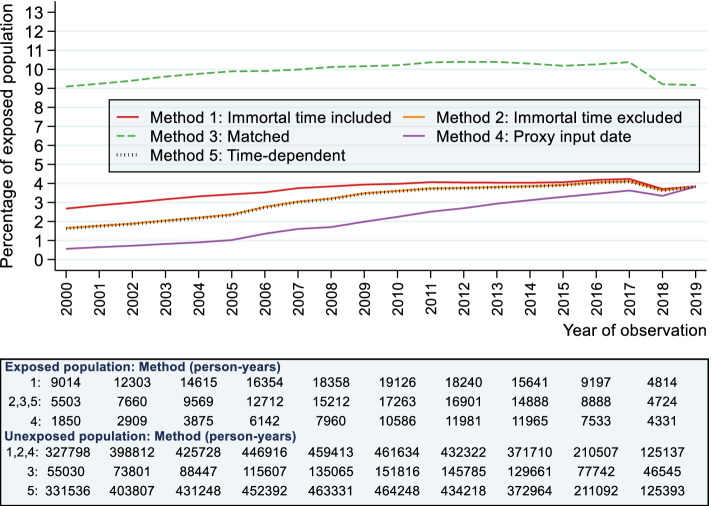
Percentage of exposed individuals by year of observation ^a, b,c^. ^a^ Method 4 can contain more than one individual where first intellectual disability diagnosis date is greater than the date of entry (e.g. a person entering the cohort in 2000 but diagnosed first with intellectual disability in 2006 enters the cohort without intellectual disabilities in 2000 and enters again with intellectual disabilities in 2006). ^b^ Please note that, as a random sample of the general population without intellectual disabilities for the comparison (unexposed) group, this graph cannot be interpreted as representing prevalence of intellectual disability. ^c^ As Method 3 involved matching on cohort entry at a 1:10 ratio by design, approximately 10% of the sample had intellectual disabilities throughout the observation period

There is also an additional problem that, by excluding immortal time from the observation period, we introduce time-related bias by forcing many of the exposed individuals to enter later than the unexposed population (Fig. 1). Under Method 2, the median length of follow-up was shorter in the exposed population (4.6 yrs. vs 5.0 yrs.; Table S3). The percentage of people with intellectual disabilities in the entire cohort (i.e. combining the cohort with and without intellectual disability) was also smaller in the first year of cohort entry period, increasing thereafter (see Fig. 4). Bias introduced from this approach is believed to be negligible if the person-years in the exposed cohort is much smaller than the person-years in the unexposed cohort [10], but this does mean that a smaller sample of people with intellectual disabilities are investigated in earlier cohort periods. The next method attempts to evaluate this using matching techniques.

### Method 3: matching cohort entry to first exposure diagnosis

Compared to only excluding immortal time before date of diagnosis (Method 2), matching on cohort entry resulted in a more balanced distribution of people in the exposed population over the observation period (Fig. 4). As expected, the life expectancy calculations for exposed individuals were identical to Method 2 (Fig. 2). Life expectancy was also similar in the unexposed group (Fig. 3), although person-time contribution was smaller because 65.5% (*n* = 641,916) of individuals from this population were discarded from the analysis because they were not matched.

### Method 4: excluding time before proxy date of entering first exposure diagnosis

Method 4, implemented under the assumption that the CPRD’s system date represented the date that the physician input the intellectual disability diagnosis, produced the lowest life expectancy estimates for people with intellectual disabilities in the earlier calendar year periods (Fig. 2). All calendar periods, except the last calendar period, showed a markedly lower life expectancy for people with intellectual disabilities than the other two methods. The graphs show that life expectancy estimates in the unexposed population for this method increased only slightly over time (Fig. 3) with life expectancy in people with intellectual disabilities showing more dramatic improvements (Fig. 2).

However, this method led to a substantial loss in person-year contributions (median follow-up period 2.2 yrs. vs 5.0 yrs) in the earlier cohort periods (Fig. 4). During the first calendar period (2000–2004), almost three-quarters of person-year contributions were lost compared with Method 1 (78.4%, *n* = 43,787 yrs) and two-thirds compared with Method 2 (64.9%, *n* = 22,281 yrs). Person-years lost gradually lowered for the subsequent calendar periods (64.1 and 53.3%, respectively, for 2005–2009, 41.2 and 35.3% for 2010–2014; 24.0 and 20.2% for 2015–2019). We can see, therefore, that people with a good prognosis (i.e. surviving long enough to have a system update at some later point) may have systematically been removed. The supplementary Fig. S2 shows the proportion of system dates that were linked to first diagnosis but were after that diagnosis. Between 45 and 80% of individuals had a linked system date after the date of their first diagnosis and between 1 and 3% of individuals (*n* = 623 in total) had a linked system date recorded after date of death or leaving the practice, which suggests that these may have been software system updates. Moreover, the use of system date as a proxy date for entering the cohort is not advocated for CPRD data because it is not sufficiently specific [31].

### Method 5: treating exposure as a time-dependent measure

The final approach, treating the exposure as a time-dependent variable, showed very similar results to Method 2 and 3 for the exposed population (Fig. 2) and marginal differences to the life expectancy calculations in the unexposed population (Fig. 3) because the additional person-year contribution from the exposed population was relatively small.

## Discussion

Through the use of electronic health records data, we demonstrate that immortal time presents a significant problem for time-to-event analyses where one or more of the exposures is a life-long condition or disability. Treating immortal time as exposure time (Method 1) led to an over-estimation of life expectancy advantages in the exposed population and could lead us to draw incorrect conclusions about survival in this population. Even when immortal time was excluded or treated as unexposed time (Methods 2, 3 and 5), some residual immortal time bias may have remained where diagnoses had been backdated to date of birth. Using proxy date of physician’s input of exposure diagnosis (Method 4) resulted in a substantial loss of person-time and subjects, and did not appear to be used in a consistent way in our data source. Our findings highlight that interpretation is key for any study where the exposure can occur after the start of the follow-up period and consideration of immortal time bias is needed to avoid drawing incorrect conclusions.

To our knowledge, this is the first time that the issue of immortal time bias has been studied in detail for life-long conditions or disabilities, although there is a wealth of literature that discusses immortal time bias in the context of pharmacoepidemiology studies. This literature largely corresponds with Methods 3 (matched) and 5 (time-dependent) in our study by recommending the control of immortal time bias through PTDM or time-dependent approaches that allow exposure status to vary [9, 10, 22]. Time-dependent analyses have also been recommended to control for the effects of immortal time bias where time of onset of certain health conditions varies, such as the menopause [3]. The reason that the issue of immortal time bias for life-long conditions/disabilities has not been considered before may be that it seems conceptually inappropriate to consider someone with a life-long condition to be ‘disease-free’ for any period of observation. However, this is likely to be the best solution for electronic health records that do not follow up individuals from birth.

The magnitude of immortal time bias is reported to be related to mean interval between date of cohort entry and date of (recording of) exposure, proportion of exposed study participants, and length of study follow-up [32]. The prevalence of intellectual disability, as diagnosed in primary care, is approximately 0.5% [33] but the current study population had a larger proportion of exposed individuals (~ 3%) because only a proportion of the unexposed population was selected for comparison. The cohort period of almost 20 years also increased the likely bias introduced by immortal time. Similarly, the choice of intellectual disability as an example may have led to substantially more immortal time bias than some other conditions or disabilities. Intellectual disability itself does not require treatment so, for administrative purposes, may not need to be entered onto GP systems if it is already known or reported in the patients’ notes. This changed in 2004, with the introduction of the Quality Outcomes Framework (QOF) and incentives to report long-term conditions including intellectual disabilities [12, 13], closely followed by policy drives to maintain practice-level intellectual disability registers in 2006 (adults) and 2014 (children) [14–16], and annual intellectual disability health checks in 2008 (adults) and 2014 (14–17 year olds) [14, 16]. We cannot identify another condition or disability where a policy drive has been so influential in changing practice in primary care. However, increased awareness is known to ‘artificially’ increase incidence of certain conditions over time, such as autism and coeliac disease [34, 35]. Our findings may also have applications for conditions where there is a delay between onset and diagnosis, such as Crohn’s disease or rare diseases [36, 37]. We also note that delays in time to diagnosis and, therefore, potential for immortal time bias may be more prevalent among people with certain characteristics, such as gender and ethnic inequalities in time to diagnosis of cancers [38, 39].

The study has a number of limitations that need to be considered. First, we only focused on one life-long condition (intellectual disabilities) so are unable to quantify the impact of immortal time on other conditions. Second, we included individuals with a diagnosis of intellectual disabilities ‘ever’ in their electronic health records before the date that the surgery was known to be up-to-standard using the CPRD’s quality indicators. Therefore, we may have included individuals with incorrect (or suspected only) diagnoses of intellectual disabilities at birth, thereby overestimating life expectancy in this population. We may also have missed individuals from surgeries that did not have computerised health record data at the time, thereby systematically excluding individuals born in earlier cohort periods who are likely to have had poorer life expectancy. We also chose to present five-year-period life expectancies to highlight our findings. We recognise that there may have been period and cohort effects during each five-year calendar period that were effectively averaged out during each calendar period. In addition, we did not have information on severity of intellectual disabilities which is a known predictor of premature mortality in this population [40], although we have no reason to believe that there has been a difference in reporting by severity over time.

We have shown that attempts to control for immortal time bias in the design or analysis stage does not guarantee unbiased results. However, treating immortal time as observation time, thereby ignoring immortal time bias completely, is not recommended as we have shown that this will lead to spurious results owing to the ‘misclassification of immortal time’ [10]. Incorporating date of assumed input of the exposure diagnosis by the physician (Method 4) may have potential in some data sources but we would also not recommend this for studies that use the CPRD owing to the apparent inconsistent use of the system date field that we used to capture date of record input and substantial loss to person-time and subjects. Studies of electronic health records where date of input of diagnosis is more reliably recorded may be considered for this purpose. This approach may also be valuable for conducting sensitivity analyses where immortal time bias is not perceived to be adequately controlled. Methods that start follow-up for exposed individuals at diagnosis (Methods 2, 3 and 5) all produce fairly similar findings and appear to solve the main theoretical problem, so any of these approaches could be adopted without a reference standard on which to compare. Although these methods all showed similar results for this study, Methods 3 (matched) and 5 (time-dependent) have conceptual advantages over Method 2 (immortal time excluded) because they do not involve conditioning on the future [41]. In other words, they allow exposed individuals to contribute to the unexposed population prior to their first diagnosis when they are still “at risk”. Finally, we recommend adding an assessment of immortal time bias as a key component of critical appraisal tools to assess the quality of observational studies with life-long conditions as exposures, particularly those that use electronic health records.

## Supplementary Information

**Additional file 1.**

## Data Availability

Data for this study were obtained from the Clinical Practice Research Datalink (CPRD), provided by the UK Medicines and Healthcare products Regulatory Agency. The authors’ licence for using these data does not allow sharing of raw data with third parties. Information about access to CPRD data is available here: https://www.cprd.com/research-applications. Researchers should contact the ISAC Secretariat at isac@cprd.com for further details.
