# Supplementary material for: Immortal time bias for life-long conditions in retrospective observational studies using electronic health records
Source: BMC Med Res Methodol. 2022 Mar 27;22:86. doi: 10.1186/s12874-022-01581-1 (PMC8962148; doi:10.1186/s12874-022-01581-1)

**Immortal time bias for life-long conditions in retrospective observational studies using electronic health records**

**Supplementary material**

**Table S1. RECORD^a^ checklist (Reporting of studies conducted using observational routinely-collected data;** [**https://www.record-statement.org/checklist.php**](https://www.record-statement.org/checklist.php)**)**

|  | **Item No.** | **STROBE items** | **Location in manuscript where items are reported** (& page number in original manuscript) | | **RECORD items** | **Location in manuscript where items are reported** | |
| --- | --- | --- | --- | --- | --- | --- | --- |
| **Title and Abstract** | | | | | | |  |
|  | 1 | (a) Indicate the study’s design with a commonly used term in the title or the abstract | 🗹 Observational electronic health record studies in **Abstract**. Source of data (CPRD) cited in **Abstract**. | p.2 | RECORD 1.1: The type of data used should be specified in the title or abstract. When possible, the name of the databases used should be included. | 🗹 Source of data (CPRD) cited in **Abstract** | p.2 |
|  |  | (b) Provide in the abstract an informative and balanced summary of what was done and what was found | 🗹 See **Abstract** | p.2 | RECORD 1.2: If applicable, the geographic region and timeframe within which the study took place should be reported in the title or abstract. | 🗹 Calendar periods (2000–2019) listed in **Abstract** | p.2 |
|  |  |  |  |  | RECORD 1.3: If linkage between databases was conducted for the study, this should be clearly stated in the title or abstract | 🗹 Linked mortality data mentioned in the **Abstract** | p.2 |
| **Introduction** | | | | | | |  |
| Background rationale | 2 | Explain the scientific background and rationale for the investigation being reported | 🗹 See **Background** | p.3 |  | | |
| Objectives | 3 | State specific objectives, including any prespecified hypotheses | 🗹 Intention to show how immortal time bias remains an issue is specified in the last paragraph of the **Background**. | p.3 |  | | |
| **Methods** | | | | | | | |
| Study Design | 4 | Present key elements of study design early in the paper | 🗹 See **Sources of Data** and **Exposure/control definitions to handling immortal time bias under five different approaches** | p.3–8 |  | | |
| Setting | 5 | Describe the setting, locations, and relevant dates, including  periods of recruitment, exposure, follow-up, and data collection | 🗹 See **Sources of Data** | p.3–4 |  | | |
| **Participants** | 6 | (a) *Cohort study* - Give the eligibility criteria, and the sources and methods of selection of participants. Describe methods of follow-up  *Case-control study* - Give the eligibility criteria, and the sources and methods of case ascertainment and control selection. Give the rationale for the choice of cases and controls  *Cross-sectional study* - Give the eligibility criteria, and the sources and methods of selection of participants | 🗹 Inclusion and data of entry/exit criteria listed in **Sources of Data.** Entry criteria varied by choice of method | p.3–4 | RECORD 6.1: The methods of study population selection (such as codes or algorithms used to identify subjects) should be listed in detail. If this is not possible, an explanation should be provided. | 🗹 All codes are listed in the supplementary material | (Supp)  p.11 |
|  |  | (b) *Cohort study* For matched studies, give matching criteria and number of exposed and unexposed  *Case-control study* - For matched studies, give matching criteria and the number of controls per case | 🗹 For method 3, matching criteria specified, how achieved and ratio of exposed to unexposed (1:10) | p.6 | RECORD 6.2: Any validation studies of the codes or algorithms used to select the population should be referenced. If validation was conducted for this study and not published elsewhere, detailed methods and results should be provided. | 🗹 Codes were identified from the literature, free-text searching of Read code descriptions and clinical opinion, as described. | (Supp)  p.11 |
|  |  |  |  |  | RECORD 6.3: If the study involved linkage of databases, consider use of a flow diagram or other graphical display to demonstrate the data linkage process, including the number of individuals with linked data at each stage. | 🗹 See supplementary material (Figure S1). | (Supp)  p.21 |
| Variables | 7 | Clearly define all outcomes, exposures, predictors, potential confounders, and effect modifiers. Give diagnostic criteria, if applicable. | 🗹 The outcome is mortality for this methodological article. Data were also stratified by exposed/unexposed status and calendar year period. However, traditional confounders were not applied for this methodological work. | p.4–8 | RECORD 7.1: A complete list of codes and algorithms used to classify exposures, outcomes, confounders, and effect modifiers should be provided. If these cannot be reported, an explanation should be provided. | 🗹 See supplementary material. | (Supp)  p.11 |
| Data sources/ measurement | 8 | For each variable of interest, give sources of data and details of methods of assessment (measurement).  Describe comparability of assessment methods if there is more than one group | 🗹 See **Data Sources**. All codes are provided in the supplementary material, including ethnicity which is used to describe for baseline characteristics. | (Supp)  p.11 |  | | |
| Bias | 9 | Describe any efforts to address potential sources of bias | 🗹 This methodological paper compares several approaches to handling/ignoring immortal time bias | p.4–8 |  | | |
| Study size | 10 | Explain how the study size was arrived at | 🗷 All of the exposed population on the CPRD meeting eligibility criteria were selected. Feasibility counts for sample size are included in the cited ISAC protocol number. | - |  | | |
| Quantitative variables | 11 | Explain how quantitative variables were handled in the analyses. If applicable, describe which groupings were chosen, and why | 🗹 Subjects were grouped into calendar year periods to assess immortal time bias effects over time | p.4 |  | | |
| Statistical methods | 12 | (a) Describe all statistical methods, including those used to control for confounding | 🗹 See **Statistical analyses** | p.8 |  | | |
|  |  | (b) Describe any methods used to examine subgroups and interactions | 🗹 Subjects were stratified by calendar period and exposure status to demonstrate the effects of immortal time bias. | p.8 |  |  |  |
|  |  | (c) Explain how missing data were addressed | 🗷 N/A for this study | - |  |  |  |
|  |  | (d) *Cohort study* - If applicable, explain how loss to follow-up was addressed  *Case-control study* - If applicable, explain how matching of cases and controls was addressed  *Cross-sectional study* - If applicable, describe analytical methods taking account of sampling strategy | 🗹 Right censoring strategy described in **Methods** (**Sources of data**) | p.4 |  |  |  |
|  |  | (e) Describe any sensitivity analyses | 🗷 N/A for this study. | - |  |  |  |
| Data access and cleaning methods |  | | | | RECORD 12.1: Authors should describe the extent to which the investigators had access to the database population used to create the study population. | 🗹 This is an established research database that has been quality assessed and checked for internal validity. Relevant papers have been cited in **Sources of Data.** | p.3 |
|  |  |  |  |  | RECORD 12.2: Authors should provide information on the data cleaning methods used in the study. | 🗹 Relevant codes have been described for this study and how they were used. Data cleaning was minimal because measurements/ results were not used for this analysis. | (Supp)  p.11 |
| Linkage |  | | | | RECORD 12.3: State whether the study included person-level, institutional-level, or other data linkage across two or more databases. The methods of linkage and methods of linkage quality evaluation should be provided. | 🗹 Person-level linkage was used for this analysis as specified in the methods (**Sources of Data**) | p.3 |
| **Results** | | | | | | | |
| Participants | 13 | (a) Report the numbers of individuals at each stage of the study (*e.g.*, numbers potentially eligible, examined for eligibility, confirmed eligible, included in the study, completing follow-up, and analysed) | 🗹 Numbers of patients in each exposure category under each method are described in the supplementary **Table S3**. Baseline characteristics are also described.  The extraction from the CPRD was based on pre-agreed criteria (e.g. linkage available and up-to-standard practices). Exclusion of patients at different stages are described in the supplementary tables. Person years at risk are also described in each of the figures. | (Supp)  p.22 | RECORD 13.1: Describe in detail the selection of the persons included in the study (*i.e.,* study population selection) including filtering based on data quality, data availability and linkage. The selection of included persons can be described in the text and/or by means of the study flow diagram. | 🗹 A description of subjects under all methods is provided in the supplementary table (**Table S3**). The exact flow of patients is also available in supplementary **Figure S1**. | (Supp)  p.21–22 |
|  |  | (b) Give reasons for non- participation at each stage. | 🗹 Where available, number of relevant individuals is shown in the relevant tables/figures and supplementary material. | (Supp)  p.22 |  |  |  |
|  |  | (c) Consider use of a flow diagram | 🗷 Not reported but available on request. | - |  |  |  |
| Descriptive data | 14 | (a) Give characteristics of study participants (*e.g.*, demographic, clinical, social) and information on exposures and potential confounders | 🗹 Baseline characteristics of the study population are shown in the supplementary table (**Table S3**). | (Supp)  p.22 |  | | |
|  |  | (b) Indicate the number of participants with missing data for each variable of interest | 🗹 Baseline characteristics (**Table S3**) | (Supp)  p.22 |  |  |  |
|  |  | (c) *Cohort study* - summarise follow-up time (*e.g.*, average and total amount) | 🗹 Baseline characteristics (**Table S3** – median and range of follow-up given) | (Supp)  p.22 |  |  |  |
| Outcome data | 15 | *Cohort study* - Report numbers of outcome events or summary measures over time  *Case-control study* - Report numbers in each exposure category, or summary measures of exposure  *Cross-sectional study* - Report numbers of outcome events or summary measures | 🗹 Baseline characteristics (**Table S3**) and person years at risk reported. | (Supp)  p.22  See figures |  | | |
|  | 16 | (a) Give unadjusted estimates and, if applicable, confounder- adjusted estimates and their precision (e.g., 95% confidence interval). Make clear which confounders were adjusted for and why they were included | 🗹 95% confidence intervals reported in the figures. | See figures |  | | |
|  |  | (b) Report category boundaries when continuous variables were categorized | 🗹 Calendar year boundaries reported. | p.10–11 |  |  |  |
|  |  | (c) If relevant, consider translating estimates of relative risk into absolute risk for a meaningful time period | 🗹 Life expectancy reported. However, this methodological paper does not seek to interpret the clinical findings but rather to discuss the issues of bias. | See figures |  |  |  |
| Other analyses | 17 | Report other analyses done— e.g., analyses of subgroups and interactions, and sensitivity analyses | N/A | - |  | | |
| **Discussion** | | | | | | | |
| Key results | 18 | Summarise key results with  reference to study objectives | 🗹 See **Discussion** | p.15 |  | | |
| Limitations | 19 | Discuss limitations of the study, taking into account sources of potential bias or imprecision.  Discuss both direction and magnitude of any potential bias | 🗹 See **Discussion** | p.16 | RECORD 19.1: Discuss the implications of using data that were not created or collected to answer the specific research question(s). Include discussion of misclassification bias, unmeasured confounding, missing data, and changing eligibility over time, as they pertain to the study being  reported. | 🗹 The article discusses immortal time bias in the context of life-long conditions and the dangers of constructing cohorts from electronic health record data that were not specifically designed for this purpose. | p.15–17 |
| Interpretation | 20 | Give a cautious overall interpretation of results considering objectives, limitations, multiplicity of analyses, results from similar studies, and other relevant evidence | 🗹 See **Discussion** | p.15–17 |  | | |
| Generalisability | 21 | Discuss the generalisability (external validity) of the study results | 🗹 See **Discussion** | p.15–17 |  | | |
| **Other Information** | | | | | | | |
| Funding | 22 | Give the source of funding and the role of the funders for the present study and, if applicable, for the original study on which the present article is based | 🗹 See **Funding** | p.21 |  | | |
| Accessibility of protocol, raw data, and programming  code |  | | | | RECORD 22.1: Authors should provide information on how to access any supplemental information such as the study protocol, raw data, or programming code. | 🗹 The CPRD’s ISAC study protocol ID is listed and available to view on-line (**Sources of Data**). Raw data are not available and an explanation is given for this in the **Availability of Data** statement. | p.21 |

Checklist is protected under Creative Commons Attribution ([CC BY](http://creativecommons.org/licenses/by/4.0/)) licence.

^a^ Reference: Benchimol EI, Smeeth L, Guttmann A, Harron K, Moher D, Petersen I, Sørensen HT, von Elm E, Langan SM, the RECORD Working Committee. The REporting of studies Conducted using Observational Routinely-collected health Data (RECORD) Statement. *PLoS Medicine* 2015; 2(10): e1001885. https://doi.org/10.1371/journal.pmed.1001885

**Table S2: Diagnostic and classification codes**

| **Primary care Read codes for intellectual disability diagnoses**  (present in either clinical, tests or referral data on the CPRD) | | | |
| --- | --- | --- | --- |
| **Read Code** | **Read Code Description** | | |
| C31yX00 | Disorder of glycoprotein metabolism, unspecified | | |
| C372000 | Hypoxanthine-guanine-phosphoribosyltransferase deficiency | | |
| C372011 | Lesch - Nyhan syndrome | | |
| C372300 | Lesch-Nyhan syndrome | | |
| C372z00 | Other disorder of purine or pyrimidine metabolism NOS | | |
| E141000 | Active disintegrative psychoses | | |
| E141100 | Residual disintegrative psychoses | | |
| E141z00 | Disintegrative psychosis NOS | | |
| Eu70000 | [X]Mld mental retard with statement no or min impairm behav | | |
| Eu70100 | [X]Mld mental retard sig impairment behav req attent/treatmt | | |
| Eu70y00 | [X]Mild mental retardation, other impairments of behaviour | | |
| Eu70z00 | [X]Mild mental retardation without mention impairment behav | | |
| Eu71000 | [X]Mod mental retard with statement no or min impairm behav | | |
| Eu71100 | [X]Mod mental retard sig impairment behav req attent/treatmt | | |
| Eu71y00 | [X]Mod retard oth behav impair | | |
| Eu71z00 | [X]Mod mental retardation without mention impairment behav | | |
| Eu72000 | [X]Sev mental retard with statement no or min impairm behav | | |
| Eu72100 | [X]Sev mental retard sig impairment behav req attent/treatmt | | |
| Eu72y00 | [X]Severe mental retardation, other impairments of behaviour | | |
| Eu72z00 | [X]Sev mental retardation without mention impairment behav | | |
| Eu73000 | [X]Profound ment retrd wth statement no or min impairm behav | | |
| Eu73100 | [X]Profound ment retard sig impairmnt behav req attent/treat | | |
| Eu73y00 | [X]Profound mental retardation, other impairments of behavr | | |
| Eu73z00 | [X]Prfnd mental retardation without mention impairment behav | | |
| Eu7y000 | [X]Oth mental retard with statement no or min impairm behav | | |
| Eu7y100 | [X]Oth mental retard sig impairment behav req attent/treatmt | | |
| Eu7yy00 | [X]Other mental retardation, other impairments of behaviour | | |
| Eu7yz00 | [X]Other mental retardation without mention impairment behav | | |
| Eu7z000 | [X]Unsp mental retard with statement no or min impairm behav | | |
| Eu7z100 | [X]Unsp mentl retard sig impairment behav req attent/treatmt | | |
| Eu7zy00 | [X]Unspecified mental retardatn, other impairments of behav | | |
| Eu7zz00 | [X]Unsp mental retardation without mention impairment behav | | |
| Eu81400 | [X]Moderate learning disability | | |
| Eu81500 | [X]Severe learning disability | | |
| Eu81600 | [X]Mild learning disability | | |
| Eu81700 | [X]Profound learning disability | | |
| Eu81800 | [X]Specific learning disability | | |
| Eu81z00 | [X]Developmental disorder of scholastic skills, unspecified | | |
| Eu81z11 | [X]Learning disability NOS | | |
| Eu81z12 | [X]Learning disorder NOS | | |
| Eu81z13 | [X]Learn acquisition disab NOS | | |
| Eu84112 | [X]Mental retardation with autistic features | | |
| Eu84200 | [X]Rett's syndrome | | |
| Eu84300 | [X]Other childhood disintegrative disorder | | |
| Eu84311 | [X]Dementia infantalis | | |
| Eu84312 | [X]Disintegrative psychosis | | |
| Eu84313 | [X]Heller's syndrome | | |
| Eu84400 | [X]Overactive disorder assoc mental retard/stereotype movts | | |
| P22yz00 | Other reduction deformity of brain NOS | | |
| PJ33100 | Deletion of long arm of chromosome 18 | | |
| PJ33111 | 18p- syndrome | | |
| PJ33200 | Deletion of short arm of chromosome 18 | | |
| PJ33211 | 18q- syndrome | | |
| PJ33300 | Smith-Magenis syndrome | | |
| PJ33400 | Jacobsen syndrome | | |
| PJ33500 | Greig cephalopolysyndactyly syndrome | | |
| PJ33700 | 3p deletion syndrome | | |
| PJ33800 | Chromosome 4q deletion syndrome | | |
| PJ33900 | Langer-Giedion syndrome | | |
| PJ33A00 | Kleefstra syndrome | | |
| PJ50000 | Trisomy 6 | | |
| PJ50100 | Trisomy 7 | | |
| PJ50200 | Trisomy 8 | | |
| PJ50300 | Trisomy 9 | | |
| PJ50400 | Trisomy 10 | | |
| PJ50500 | Trisomy 11 | | |
| PJ50600 | Trisomy 12 | | |
| PJ50700 | Other trisomy C syndromes | | |
| PJ50800 | Trisomy 22 | | |
| PJ50w00 | Whole chromosome trisomy, meitotic nondisjunction | | |
| PJ50x00 | Whole chromosome trisomy, mosaicism | | |
| PJ50x11 | Whole chromosome trisomy, mitotic nondisjunction | | |
| PJ50y00 | Other specified whole chromosome trisomy syndrome | | |
| PJ50z00 | Whole chromosome trisomy syndrome NOS | | |
| PJ51000 | Major partial trisomy | | |
| PJ51100 | Minor partial trisomy | | |
| PJ51200 | 10q partial trisomy syndrome | | |
| PJ51300 | Trisomy 4p syndrome | | |
| PJ51400 | Trisomy 9p syndrome | | |
| PJ51500 | 15q partial trisomy syndrome | | |
| PJ51z00 | Partial trisomy syndrome NOS | | |
| PJ52300 | Triploidy | | |
| PJ52400 | Polyploidy | | |
| PJ52z00 | Trisomy of autosomes NEC NOS | | |
| PJyy200 | Fragile X chromosome | | |
| PJyy400 | Fragile X syndrome | | |
| PKy6100 | Cockayne syndrome | | |
| PKy9300 | Prader - Willi syndrome | | |
| PKyz.11 | Cockayne's syndrome | | |
| PKyz511 | Angelman syndrome | | |
| PKyz700 | Angelman's syndrome | | |
| Pyu0200 | [X]Other reduction deformities of brain | | |
| Pyu0300 | [X]Other specified congenital malformations of brain | | |
| PyuA000 | [X]Oth specif trisomies & partial trisomies of autosomes | | |
| R034y11 | [D]Global retardation | | |
| ZL1B500 | Under care of psychiatrist for mental handicap | | |
| 918e.00 | On learning disability register | | |
| C031.00 | Goitrous cretin | | |
| C0A..00 | Congenital iodine deficiency syndrome | | |
| C0A0.00 | Congenital iodine-deficiency syndrome, neurological type | | |
| C0A1.00 | Congenital iodine-deficiency syndrome, myxoedematous type | | |
| C301.00 | Phenylketonuria | | |
| C372.00 | Other disorders of purine and pyrimidine metabolism | | |
| C377.00 | Disorders of glycoprotein metabolism | | |
| E141.00 | Disintegrative psychosis | | |
| E3..00 | Mental retardation | | |
| E30..00 | Mild mental retardation, IQ in range 50-70 | | |
| E31..00 | Other specified mental retardation | | |
| E310.00 | Moderate mental retardation, IQ in range 35-49 | | |
| E311.00 | Severe mental retardation, IQ in range 20-34 | | |
| E312.00 | Profound mental retardation with IQ less than 20 | | |
| E31z.00 | Other specified mental retardation NOS | | |
| E3y..00 | Other specified mental retardation | | |
| E3z..00 | Mental retardation NOS | | |
| Eu7..00 | [X]Mental retardation | | |
| Eu70.00 | [X]Mild mental retardation | | |
| Eu71.00 | [X]Moderate mental retardation | | |
| Eu72.00 | [X]Severe mental retardation | | |
| Eu73.00 | [X]Profound mental retardation | | |
| Eu7y.00 | [X]Other mental retardation | | |
| Eu7z.00 | [X]Unspecified mental retardation | | |
| P01..00 | Craniorachischisis | | |
| P02..00 | Iniencephaly | | |
| P22..00 | Reduction deformities of brain | | |
| P224.00 | Arhinencephaly | | |
| P225.00 | Holoprosencephaly | | |
| P22y.00 | Other specified reduction deformities of brain | | |
| P22z.00 | Reduction deformities of brain NOS | | |
| PJ0..00 | Down's syndrome - trisomy 21 | | |
| PJ00.00 | Trisomy 21, meiotic nondisjunction | | |
| PJ02.00 | Trisomy 21, translocation | | |
| PJ0z.00 | Down's syndrome NOS | | |
| PJ1..00 | Patau's syndrome - trisomy 13 | | |
| PJ10.00 | Trisomy 13, meiotic nondisjunction | | |
| PJ11.00 | Trisomy 13, mosaicism | | |
| PJ12.00 | Trisomy 13, translocation | | |
| PJ1z.00 | Patau's syndrome NOS | | |
| PJ2..00 | Edward's syndrome - trisomy 18 | | |
| PJ20.00 | Trisomy 18, meiotic nondisjunction | | |
| PJ21.00 | Trisomy 18, mosaicism | | |
| PJ22.00 | Trisomy 18, translocation | | |
| PJ2z.00 | Edward's syndrome NOS | | |
| PJ30.00 | Antimongolism syndrome | | |
| PJ31.00 | Cri-du-chat syndrome | | |
| PJ32.00 | Deletion of short arm of chromosome 4 | | |
| PJ3z.00 | Monosomies and deletions from the autosomes NOS | | |
| PJ50.00 | Whole chromosome trisomy syndromes | | |
| PJ51.00 | Partial trisomy syndromes | | |
| PJ52.00 | Trisomies of autosomes NEC | | |
| PJ9..00 | Mowat-Wilson syndrome | | |
| PK5..00 | Tuberous sclerosis | | |
| PKy4.00 | William syndrome | | |
| ZS34.00 | Developmental disorder of scholastic skill | | |
| E3...00 | Mental retardation | | |
| C03..11 | Cretinism | | |
| C372.11 | Lesch - Nyhan syndrome | | |
| E141.11 | Heller's syndrome | | |
| E30..11 | Educationally subnormal | | |
| E310.11 | Imbecile | | |
| E312.11 | Idiocy | | |
| Eu70.11 | [X]Feeble-mindedness | | |
| Eu71.11 | [X]Moderate mental subnormality | | |
| Eu72.11 | [X]Severe mental subnormality | | |
| Eu73.11 | [X]Profound mental subnormality | | |
| Eu7z.11 | [X]Mental deficiency NOS | | |
| PJ0..11 | Mongolism | | |
| PJ01.11 | Trisomy 21, mitotic nondisjunction | | |
| PJ02.11 | Partial trisomy 21 in Down's syndrome | | |
| PJ0z.11 | Trisomy 21 NOS | | |
| PJ11.11 | Trisomy 13, mitotic nondisjunction | | |
| PJ12.11 | Partial trisomy 13 in Patau's syndrome | | |
| PJ1z.11 | Trisomy 13 NOS | | |
| PJ21.11 | Trisomy 18, mitotic nondisjunction | | |
| PJ22.11 | Partial trisomy 18 in Edward's syndrome | | |
| PJ2z.11 | TRISOMY 18 NOS | | |
| PJ30.11 | Deletion of long arm of chromosome 21 | | |
| PJ31.11 | Deletion of short arm of chromosome 5 | | |
| PJ32.11 | Wolff - Hirschorn syndrome | | |
| PKy0.11 | Prader-Willi Syndrome | | |
| ZS34.11 | Learning disability | | |
| C03z.12 | Cretinism | | |
| E30..12 | Feeble-minded | | |
| Eu70.12 | [X]Mild mental subnormality | | |
| Eu7z.12 | [X]Mental subnormality NOS | | |
| PJ0..12 | Trisomy 21 | | |
| PKy0.12 | Prader-Willi syndrome | | |
| E30..13 | Moron | | |
| PJ0..13 | Trisomy 22 | | |
| 8Ce6.00 | Preferred place of care - learning disability unit | | |
| 9HB..00 | Learning disabilities administration status | | |
| 9HB0.00 | Learning disabilities health action plan declined | | |
| 9HB1.00 | Learning disabilities health action plan offered | | |
| 9HB2.00 | Learning disabilities health action plan reviewed | | |
| 9HB3.00 | Learning disabilities health assessment | | |
| 9HB4.00 | Learning disabilities health action plan completed | | |
| 9HB5.00 | Learning disabilities annual health assessment | | |
| 9HB6.00 | Learning disabilities annual health assessment declined | | |
| 9HB7.00 | Did not attend learning disabilities annual health assessmnt | | |
| 9hL..00 | Exception reporting: learning disability quality indicators | | |
| 9hL0.00 | Exc learn disability quality indicators: informed dissent | | |
| 9hL1.00 | Exc learn disability quality indicators: patient unsuitable | | |
| 9mA..00 | Learning disability annual health check invitation | | |
| 9mA0.00 | Learning disability annual health check verbal invitation | | |
| 9mA1.00 | Learning disability annual health check telephone invitation | | |
| 9mA2.00 | Learning disability annual health check letter invitation | | |
| 9mA2000 | Learning disability annual health check invtation 1st letter | | |
| 9mA2100 | Learning disability annual health check invtation 2nd letter | | |
| 9mA2200 | Learning disability annual health check invtation 3rd letter | | |
| 9Nh4.00 | Under care of community learning disability team | | |
| 9HB6.11 | Learning disabilities annual health check declined | | |
| 9HB7.11 | Did not attend learning disabilities annual health check | | |
| 13Z3.00 | Low I.Q. | | |
| 69DB.00 | Learning disability health examination | | |
| 94Z9.00 | Preferred place of death: learning disability unit | | |
| 6664 | Mental handicap problem | | |
| **Primary care Read codes for ethnicity status** | | | |
| **Read Code** | | **Read code description** | **Ethnic group** |
| 916E.00 | | Patient ethnicity unknown | Ethnic group not specified |
| 9i...00 | | Ethnic category - 2001 census | Ethnic group not specified |
| 9i0..00 | | British or mixed British - ethnic category 2001 census | Ethnic group not specified |
| 9i00.00 | | White British - ethnic category 2001 census | White |
| 9i1..00 | | Irish - ethnic category 2001 census | White |
| 9i10.00 | | White Irish - ethnic category 2001 census | White |
| 9i2..00 | | Other White background - ethnic category 2001 census | White |
| 9i20.00 | | English - ethnic category 2001 census | White |
| 9i21.00 | | Scottish - ethnic category 2001 census | White |
| 9i22.00 | | Welsh - ethnic category 2001 census | White |
| 9i23.00 | | Cornish - ethnic category 2001 census | White |
| 9i24.00 | | Northern Irish - ethnic category 2001 census | White |
| 9i25.00 | | Ulster Scots - ethnic category 2001 census | White |
| 9i26.00 | | Cypriot (part not stated) - ethnic category 2001 census | White |
| 9i27.00 | | Greek - ethnic category 2001 census | White |
| 9i28.00 | | Greek Cypriot - ethnic category 2001 census | White |
| 9i29.00 | | Turkish - ethnic category 2001 census | White |
| 9i2A.00 | | Turkish Cypriot - ethnic category 2001 census | White |
| 9i2B.00 | | Italian - ethnic category 2001 census | White |
| 9i2C.00 | | Irish Traveller - ethnic category 2001 census | White |
| 9i2D.00 | | Traveller - ethnic category 2001 census | White |
| 9i2E.00 | | Gypsy/Romany - ethnic category 2001 census | White |
| 9i2F.00 | | Polish - ethnic category 2001 census | White |
| 9i2G.00 | | Baltic Estonian/Latvian/Lithuanian - ethn categ 2001 census | White |
| 9i2H.00 | | Commonwealth (Russian) Indep States - ethn categ 2001 census | White |
| 9i2J.00 | | Kosovan - ethnic category 2001 census | White |
| 9i2K.00 | | Albanian - ethnic category 2001 census | White |
| 9i2L.00 | | Bosnian - ethnic category 2001 census | White |
| 9i2M.00 | | Croatian - ethnic category 2001 census | White |
| 9i2N.00 | | Serbian - ethnic category 2001 census | White |
| 9i2P.00 | | Other republics former Yugoslavia - ethnic categ 2001 census | White |
| 9i2Q.00 | | Mixed Irish and other White - ethnic category 2001 census | White |
| 9i2R.00 | | Oth White European/European unsp/Mixed European 2001 census | White |
| 9i2S.00 | | Other mixed White - ethnic category 2001 census | White |
| 9i2T.00 | | Other White or White unspecified ethnic category 2001 census | White |
| 9i3..00 | | White and Black Caribbean - ethnic category 2001 census | Mixed |
| 9i4..00 | | White and Black African - ethnic category 2001 census | Mixed |
| 9i5..00 | | White and Asian - ethnic category 2001 census | Mixed |
| 9i6..00 | | Other Mixed background - ethnic category 2001 census | Mixed |
| 9i60.00 | | Black and Asian - ethnic category 2001 census | Mixed |
| 9i61.00 | | Black and Chinese - ethnic category 2001 census | Mixed |
| 9i62.00 | | Black and White - ethnic category 2001 census | Mixed |
| 9i63.00 | | Chinese and White - ethnic category 2001 census | Mixed |
| 9i64.00 | | Asian and Chinese - ethnic category 2001 census | Mixed |
| 9i65.00 | | Other Mixed or Mixed unspecified ethnic category 2001 census | Mixed |
| 9i7..00 | | Indian or British Indian - ethnic category 2001 census | S.Asian |
| 9i8..00 | | Pakistani or British Pakistani - ethnic category 2001 census | S.Asian |
| 9i9..00 | | Bangladeshi or British Bangladeshi - ethn categ 2001 census | S.Asian |
| 9iA..00 | | Other Asian background - ethnic category 2001 census | S.Asian |
| 9iA1.00 | | Punjabi - ethnic category 2001 census | S.Asian |
| 9iA2.00 | | Kashmiri - ethnic category 2001 census | S.Asian |
| 9iA3.00 | | East African Asian - ethnic category 2001 census | S.Asian |
| 9iA4.00 | | Sri Lankan - ethnic category 2001 census | Other ethnic group |
| 9iA5.00 | | Tamil - ethnic category 2001 census | Other ethnic group |
| 9iA6.00 | | Sinhalese - ethnic category 2001 census | Other ethnic group |
| 9iA7.00 | | Caribbean Asian - ethnic category 2001 census | Mixed |
| 9iA8.00 | | British Asian - ethnic category 2001 census | S.Asian |
| 9iA9.00 | | Mixed Asian - ethnic category 2001 census | S.Asian |
| 9iAA.00 | | Other Asian or Asian unspecified ethnic category 2001 census | S.Asian |
| 9iB..00 | | Caribbean - ethnic category 2001 census | Black |
| 9iC..00 | | African - ethnic category 2001 census | Black |
| 9iD..00 | | Other Black background - ethnic category 2001 census | Black |
| 9iD0.00 | | Somali - ethnic category 2001 census | Black |
| 9iD1.00 | | Nigerian - ethnic category 2001 census | Black |
| 9iD2.00 | | Black British - ethnic category 2001 census | Black |
| 9iD3.00 | | Mixed Black - ethnic category 2001 census | Black |
| 9iD4.00 | | Other Black or Black unspecified ethnic category 2001 census | Black |
| 9iE..00 | | Chinese - ethnic category 2001 census | Other ethnic group |
| 9iF..00 | | Other - ethnic category 2001 census | Other ethnic group |
| 9iF0.00 | | Vietnamese - ethnic category 2001 census | Other ethnic group |
| 9iF1.00 | | Japanese - ethnic category 2001 census | Other ethnic group |
| 9iF2.00 | | Filipino - ethnic category 2001 census | Other ethnic group |
| 9iF3.00 | | Malaysian - ethnic category 2001 census | Other ethnic group |
| 9iF4.00 | | Buddhist - ethnic category 2001 census | Other ethnic group |
| 9iF5.00 | | Hindu - ethnic category 2001 census | Other ethnic group |
| 9iF6.00 | | Jewish - ethnic category 2001 census | Other ethnic group |
| 9iF7.00 | | Muslim - ethnic category 2001 census | Other ethnic group |
| 9iF8.00 | | Sikh - ethnic category 2001 census | Other ethnic group |
| 9iF9.00 | | Arab - ethnic category 2001 census | Other ethnic group |
| 9iFA.00 | | North African - ethnic category 2001 census | Other ethnic group |
| 9iFB.00 | | Mid East (excl Israeli, Iranian & Arab) - eth cat 2001 cens | Other ethnic group |
| 9iFC.00 | | Israeli - ethnic category 2001 census | Other ethnic group |
| 9iFD.00 | | Iranian - ethnic category 2001 census | Other ethnic group |
| 9iFE.00 | | Kurdish - ethnic category 2001 census | Other ethnic group |
| 9iFF.00 | | Moroccan - ethnic category 2001 census | Other ethnic group |
| 9iFG.00 | | Latin American - ethnic category 2001 census | Other ethnic group |
| 9iFH.00 | | South and Central American - ethnic category 2001 census | Other ethnic group |
| 9iFJ.00 | | Mauritian/Seychellois/Maldivian/St Helena eth cat 2001census | Other ethnic group |
| 9iFK.00 | | Any other group - ethnic category 2001 census | Other ethnic group |
| 9iG..00 | | Ethnic category not stated - 2001 census | Ethnic group not specified |
| 9S...00 | | Ethnic groups (1991 census) | Ethnic group not specified |
| 9S1..00 | | White | White |
| 9S10.00 | | White British | White |
| 9S11.00 | | White Irish | White |
| 9S12.00 | | Other white ethnic group | White |
| 9S13.00 | | White Scottish | White |
| 9S14.00 | | Other white British ethnic group | White |
| 9S2..00 | | Black Caribbean | Black |
| 9S3..00 | | Black African | Black |
| 9S4..00 | | Black, other, non-mixed origin | Black |
| 9S41.00 | | Black British | Black |
| 9S42.00 | | Black Caribbean/W.I./Guyana | Black |
| 9S42.11 | | Black Caribbean | Black |
| 9S42.12 | | Black West Indian | Black |
| 9S42.13 | | Black Guyana | Black |
| 9S43.00 | | Black N African/Arab/Iranian | Black |
| 9S43.11 | | Black North African | Black |
| 9S43.12 | | Black Arab | Black |
| 9S43.13 | | Black Iranian | Black |
| 9S44.00 | | Black - other African country | Black |
| 9S45.00 | | Black E Afric Asia/Indo-Caribb | Mixed |
| 9S45.11 | | Black East African Asian | Mixed |
| 9S45.12 | | Black Indo-Caribbean | Mixed |
| 9S46.00 | | Black Indian sub-continent | Mixed |
| 9S47.00 | | Black - other Asian | Mixed |
| 9S48.00 | | Black Black - other | Black |
| 9S5..00 | | Black - other, mixed | Black |
| 9S51.00 | | Other Black - Black/White orig | Mixed |
| 9S52.00 | | Other Black - Black/Asian orig | Mixed |
| 9S6..00 | | Indian | S.Asian |
| 9S7..00 | | Pakistani | S.Asian |
| 9S8..00 | | Bangladeshi | S.Asian |
| 9S9..00 | | Chinese | Other ethnic group |
| 9SA..00 | | Other ethnic non-mixed (NMO) | Other ethnic group |
| 9SA1.00 | | Brit. ethnic minor. spec.(NMO) | Other ethnic group |
| 9SA2.00 | | Brit. ethnic minor. unsp (NMO) | Other ethnic group |
| 9SA3.00 | | Caribbean I./W.I./Guyana (NMO) | Black |
| 9SA3.11 | | Caribbean Island (NMO) | Black |
| 9SA3.12 | | West Indian (NMO) | Black |
| 9SA3.13 | | Guyana (NMO) | Black |
| 9SA4.00 | | N African Arab/Iranian (NMO) | Other ethnic group |
| 9SA4.11 | | North African Arab (NMO) | Other ethnic group |
| 9SA4.12 | | Iranian (NMO) | Other ethnic group |
| 9SA5.00 | | Other African countries (NMO) | Black |
| 9SA6.00 | | E Afric Asian/Indo-Carib (NMO) | Mixed |
| 9SA6.11 | | East African Asian (NMO) | S.Asian |
| 9SA7.00 | | Indian sub-continent (NMO) | S.Asian |
| 9SA8.00 | | Other Asian (NMO) | S.Asian |
| 9SA9.00 | | Irish (NMO) | White |
| 9SAA.00 | | Greek/Greek Cypriot (NMO) | White |
| 9SAA.11 | | Greek (NMO) | White |
| 9SAA.12 | | Greek Cypriot (NMO) | White |
| 9SAB.00 | | Turkish/Turkish Cypriot (NMO) | White |
| 9SAB.11 | | Turkish (NMO) | White |
| 9SAB.12 | | Turkish Cypriot (NMO) | White |
| 9SAC.00 | | Other European (NMO) | White |
| 9SAD.00 | | Other ethnic NEC (NMO) | Other ethnic group |
| 9SB..00 | | Other ethnic, mixed origin | Mixed |
| 9SB1.00 | | Other ethnic, Black/White orig | Mixed |
| 9SB2.00 | | Other ethnic, Asian/White orig | Mixed |
| 9SB3.00 | | Other ethnic, mixed white orig | White |
| 9SB4.00 | | Other ethnic, other mixed orig | Mixed |
| 9SB5.00 | | Black Caribbean and White | Mixed |
| 9SB6.00 | | Black African and White | Mixed |
| 9SC..00 | | Vietnamese | Other ethnic group |
| 9SD..00 | | Ethnic group not given - patient refused | Ethnic group not specified |
| 9SE..00 | | Ethnic group not recorded | Ethnic group not specified |
| 9SG..00 | | Other black ethnic group | Black |
| 9SH..00 | | Other Asian ethnic group | S.Asian |
| 9SI..00 | | Irish traveller | White |
| 9SJ..00 | | Other ethnic group | Other ethnic group |
| 9SZ..00 | | Ethnic groups (census) NOS | Ethnic group not specified |
| 9T...00 | | Ethnicity and other related nationality data | Ethnic group not specified |
| 9t0..00 | | Ethnic category - 2011 census England and Wales | Ethnic group not specified |
| 9t00.00 | | White:Eng/Welsh/Scot/NI/Brit - England and Wales 2011 census | White |
| 9t01.00 | | White: Irish - England and Wales ethnic category 2011 census | White |
| 9t02.00 | | White: Gypsy/Irish Traveller - Eng+Wales eth cat 2011 census | White |
| 9t03.00 | | White: other White backgrd- Eng+Wales ethnic cat 2011 census | White |
| 9t04.00 | | Mixed: White+Black Caribbean - Eng+Wales eth cat 2011 census | Mixed |
| 9t05.00 | | Mixed: White+Black African - Eng+Wales eth cat 2011 census | Mixed |
| 9t06.00 | | Mixed: White+Asian - Eng+Wales ethnic category 2011 census | Mixed |
| 9t08.00 | | Asian/Asian Brit: Indian - Eng+Wales ethnic cat 2011 census | S.Asian |
| 9t09.00 | | Asian/Asian British:Pakistani- Eng+Wales eth cat 2011 census | S.Asian |
| 9t0A.00 | | Asian/Asian Brit: Bangladeshi- Eng+Wales eth cat 2011 census | S.Asian |
| 9t0B.00 | | Asian/Asian Brit: Chinese - Eng+Wales ethnic cat 2011 census | Other ethnic group |
| 9t0C.00 | | Asian/Asian Brit: other Asian- Eng+Wales eth cat 2011 census | S.Asian |
| 9t0D.00 | | Black/African/Carib/Black Brit: African- Eng+Wales 2011 cens | Black |
| 9t0E.00 | | Black/African/Caribbn/Black Brit: Caribbean - Eng+Wales 2011 | Black |
| 9t0F.00 | | Black/Afr/Carib/Black Brit: other Black- Eng+Wales 2011 cens | Black |
| 9t0G.00 | | Other ethnic group: Arab - Eng+Wales ethnic cat 2011 census | Other ethnic group |
| 9t0H.00 | | Other ethnic: any other grp - Eng+Wales eth cat 2011 census | Other ethnic group |
| 9T1..00 | | New Zealand ethnic groups | White |
| 9t12.00 | | Mixed: White and Black Caribbean - NI ethnic cat 2011 census | Mixed |
| 9t13.00 | | Mixed: White and Black African - NI ethnic cat 2011 census | Mixed |
| 9t14.00 | | Mixed: White and Asian - NI ethnic category 2011 census | Mixed |
| 9t15.00 | | Mixed: other Mixed/multiple ethnic backgrd - NI 2011 census | Mixed |
| 9t16.00 | | Asian or Asian British: Indian - NI ethnic cat 2011 census | S.Asian |
| 9t17.00 | | Asian/Asian British: Pakistani - NI ethnic cat 2011 census | S.Asian |
| 9t18.00 | | Asian/Asian British: Bangladeshi - NI ethnic cat 2011 census | S.Asian |
| 9t19.00 | | Asian/Asian British: Chinese - NI ethnic cat 2011 census | Other ethnic group |
| 9T1A.00 | | Other Pacific ethnic group | Other ethnic group |
| 9t1E.00 | | Other ethnic group: Arab - NI ethnic category 2011 census | Other ethnic group |
| 9t1F.00 | | Other ethnic group: any other grp- NI ethnic cat 2011 census | Other ethnic group |
| 9T1Y.00 | | Other New Zealand ethnic group | White |
| 9T1Z.00 | | New Zealand ethnic group NOS | White |
| 9t2..00 | | Ethnic category - 2011 census Scotland | Ethnic group not specified |
| 9t20.00 | | White: Scottish - Scotland ethnic category 2011 census | White |
| 9t21.00 | | White: other British - Scotland ethnic category 2011 census | White |
| 9t22.00 | | White: Irish - Scotland ethnic category 2011 census | White |
| 9t24.00 | | White: Polish - Scotland ethnic category 2011 census | White |
| 9t25.00 | | White: other White ethnic grp- Scotland ethnic cat 2011 cens | White |
| 9t26.00 | | Mixed/multiple ethnic grps: any- Scot ethnic cat 2011 census | Mixed |
| 9t27.00 | | Asian: Pakistani/Pakistani Scot/Pakistani Brit- Scot 2011 | S.Asian |
| 9t28.00 | | Asian: Indian, Indian Scot/Indian Brit- Scotland 2011 census | S.Asian |
| 9t29.00 | | Bangladeshi, Bangladeshi Scot or Bangladeshi Brit- Scot 2011 | S.Asian |
| 9t2A.00 | | Asian: Chinese - Scotland ethnic category 2011 census | Other ethnic group |
| 9t2B.00 | | Asian: other Asian group - Scotland ethnic cat 2011 census | S.Asian |
| 9t2C.00 | | African: African/African Scot/African Brit - Scotland 2011 | Black |
| 9t2D.00 | | African: any other African - Scotland ethnic cat 2011 census | Black |
| 9t2G.00 | | Carib/Black: any other Black/Caribbean grp - Scotland 2011 | Black |
| 9t2H.00 | | Other ethnic grp: Arab/Arab Scot/Arab British- Scotland 2011 | Other ethnic group |
| 9t2J.00 | | Other ethnic grp: any other ethnic grp- Scotland 2011 census | Other ethnic group |
| **Free-text field from Hospital Episode Statistics’ patient file** | | | |
| **Free text** | | **Ethnic group** | |
| Bangladeshi | | S.Asian | |
| Bl_Afric | | Black | |
| Bl_Carib | | Black | |
| Bl_Other | | Black | |
| Chinese | | Other ethnic group | |
| Indian | | S.Asian | |
| Mixed | | Mixed | |
| Oth_Asian | | S.Asian | |
| Other | | Other ethnic group | |
| Pakistani | | S.Asian | |
| Unknown | | Ethnic group not specified | |
| White | | White | |

**Figure S1: Data flow diagram of selection of exposed and unexposed individuals for this study^a^**

**
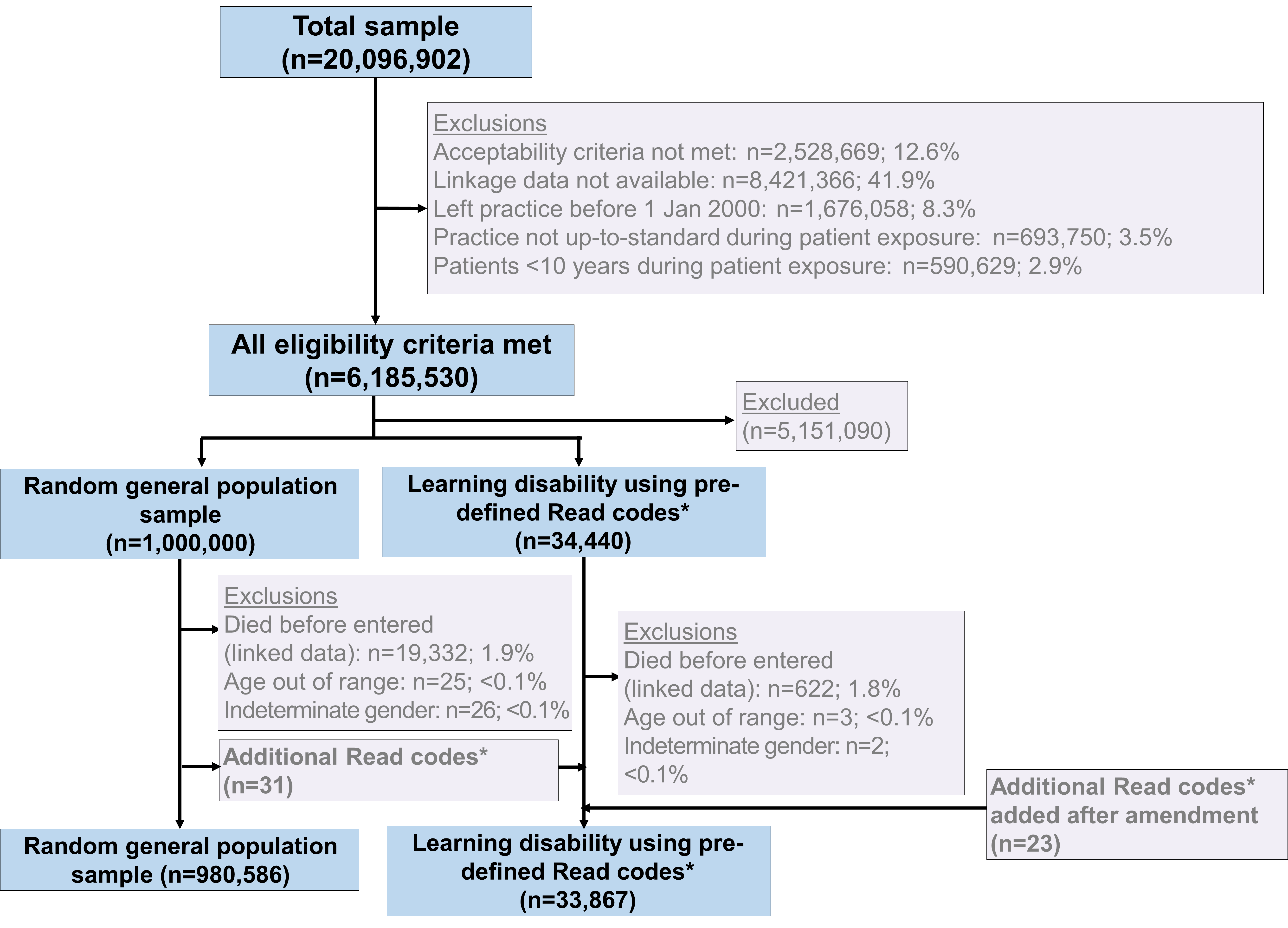
**

* Additional Read codes for Angelman and Cockayne syndrome added

^a^ An unstratified random sample was selected. Age (median 34.73yrs vs 34.74yrs), gender (48.39% vs 48.36%), proportion of deaths (estimated from CPRD data prior to deaths linkage data availability; 7.12% vs 7.13%), and length of time in the cohort (also estimated prior to linkage data availability; 5.10 vs 5.09yrs) for the selected vs entire population were compared to ensure that no obvious differences were evident.

**Table S3: Baseline characteristics of the study population using different methods to handling immortal time bias**

| **CHARACTERISTICS** | **EXPOSED** | | **NON-EXPOSED** | |
| --- | --- | --- | --- | --- |
|  | **People with intellectual disabilities**  **N (%) / median (range)** | | **People without intellectual disabilities**  **N (%) / median (range)** | |
| **Method 1** | | | | |
| Total | 33,867 | (100.00) | 980,586 | (100.00) |
| Age (years): | 29.0 | (10–102) | 34.0 | (10–108) |
| Gender: Male | 19,077 | (56.3) | 474,663 | (48.4) |
| Female | 14,790 | (43.7) | 505,923 | (51.6) |
| Ethnic group: White | 26,131 | (77.2) | 646,105 | (65.9) |
| South Asian | 774 | (2.3) | 31,384 | (3.2) |
| Black | 756 | (2.2) | 25,006 | (2.6) |
| Other ethnic group | 898 | (2.7) | 38,705 | (4.0) |
| Ethnic group not specified | 5307 | (15.7) | 239,386 | (24.4) |
| Length of observation time (years) | 6.5 | (<0.1–19.7) | 5.0 | (<0.1–19.7) |
| **Method 2** | | | | |
| Total | 33,867 | (100.00) | 980,586 | (100.00) |
| Age (years): | 31.0 | (10–102) | 34.0 | (10–108) |
| Gender: Male | 19,077 | (56.3) | 474,663 | (48.4) |
| Female | 14,790 | (43.7) | 505,923 | (51.6) |
| Ethnic group: White | 26,131 | (77.2) | 646,105 | (65.9) |
| South Asian | 774 | (2.3) | 31,384 | (3.2) |
| Black | 756 | (2.2) | 25,006 | (2.6) |
| Other ethnic group | 898 | (2.7) | 38,705 | (4.0) |
| Ethnic group not specified | 5307 | (15.7) | 239,386 | (24.4) |
| Length of observation time (years) | 4.6 | (<0.1–19.7) | 5.0 | (<0.1–19.7) |
| **Method 3^a^** | | | | |
| Total | 33,867 | (100.00) | 338,670 | (100.00) |
| Age (years): | 31.0 | (10–102) | 34.0 | (10–108) |
| Gender: Male | 19,077 | (56.3) | 161,724 | (47.8) |
| Female | 14,790 | (43.7) | 176,946 | (52.3) |
| Ethnic group: White | 26,131 | (77.2) | 221,859 | (65.5) |
| South Asian | 774 | (2.3) | 14,075 | (4.2) |
| Black | 756 | (2.2) | 10,681 | (3.2) |
| Other ethnic group | 898 | (2.7) | 17,038 | (5.0) |
| Ethnic group not specified | 5307 | (15.7) | 75,017 | (22.2) |
| Length of observation time (years) | 4.6 | (<0.1–19.7) | 3.6 | (<0.1–19.7) |
| **Method 4^b^** | | | | |
| Total | 33,244 | (100.00) | 980,586 | (100.00) |
| Age (years): | 33.0 | (10–106) | 34.0 | (10–108) |
| Gender: Male | 18,738 | (56.4) | 474,663 | (48.4) |
| Female | 14,506 | (43.6) | 505,923 | (51.6) |
| Ethnic group: White | 25,653 | (77.2) | 646,105 | (65.9) |
| South Asian | 766 | (2.3) | 31,384 | (3.2) |
| Black | 745 | (2.2) | 25,006 | (2.6) |
| Other ethnic group | 888 | (2.7) | 38,705 | (4.0) |
| Ethnic group not specified | 5191 | (15.6) | 239,386 | (24.4) |
| Length of observation time (years) | 2.2 | (<0.1–19.6) | 5.0 | (<0.1–19.7) |
| **Method 5^c^** | | | | |
| Total | 33,867 | (100.00) | 991,879 | (100.00) |
| Age (years): | 31.0 | (10–102) | 34.0 | (10–108) |
| Gender: Male | 19,077 | (56.3) | 481,223 | (48.5) |
| Female | 14,790 | (43.7) | 510,656 | (51.5) |
| Ethnic group: White | 26,131 | (77.2) | 654,776 | (66.0) |
| South Asian | 774 | (2.3) | 31,649 | (3.2) |
| Black | 756 | (2.2) | 25,289 | (2.6) |
| Other ethnic group | 898 | (2.7) | 39,009 | (3.9) |
| Ethnic group not specified | 5307 | (15.7) | 241,156 | (24.3) |
| Length of observation time (years) | 4.6 | (<0.1–19.7) | 5.0 | (<0.1–19.7) |

^a^ *n*=641,916 individuals from the unexposed population were discarded under Method 3 because they were not matched

^b^ *n*=623 individuals from the exposed population were excluded from this analysis under Method 4 because they entered on or after they were censored/died (i.e. the system date was updated for the first diagnosis after the individual had left the practice)

^c^ Individuals could contribute to both the exposed and unexposed populations under Method 5, as reflected in the baseline values

**Figure S2: Percentage of exposed individuals with a system date (proxy date of input by the physician) after date of first diagnosis, by calendar year**


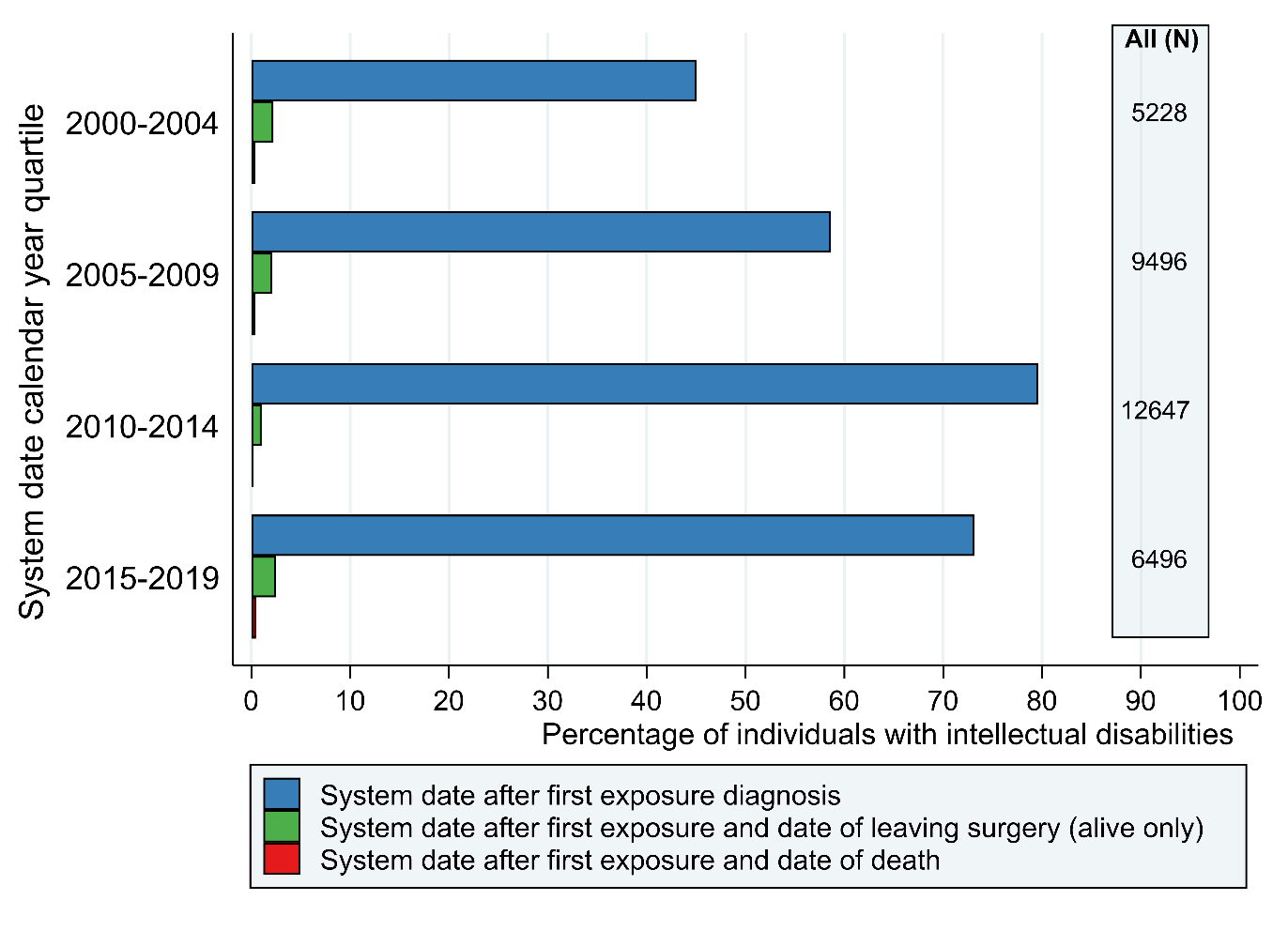

Supplement: Supplementary file 1 — Additional file 1. [file 12874_2022_1581_MOESM1_ESM.docx]
